# Engineering vesicle trafficking improves the extracellular activity and surface display efficiency of cellulases in *Saccharomyces cerevisiae*

**DOI:** 10.1186/s13068-017-0738-8

**Published:** 2017-02-27

**Authors:** Hongting Tang, Meihui Song, Yao He, Jiajing Wang, Shenghuan Wang, Yu Shen, Jin Hou, Xiaoming Bao

**Affiliations:** 1 The School of Life Science, State Key Laboratory of Microbial Technology, Shandong University, Jinan, 250100 China; 2 Shandong Provincial Key Laboratory of Microbial Engineering, Qi Lu University of Technology, Jinan, 250353 China

**Keywords:** *Saccharomyces cerevisiae*, Cellulases, Surface display protein, Vesicle trafficking, Secretory pathway

## Abstract

**Background:**

Cellulase expression via extracellular secretion or surface display in *Saccharomyces cerevisiae* is one of the most frequently used strategies for a consolidated bioprocess (CBP) of cellulosic ethanol production. However, the inefficiency of the yeast secretory pathway often results in low production of heterologous proteins, which largely limits cellulase secretion or display.

**Results:**

In this study, the components of the vesicle trafficking from the endoplasmic reticulum (ER) to the Golgi and from the Golgi to the plasma membrane, involved in vesicle budding, tethering and fusion, were over-expressed in *Clostridium thermocellum* endoglucanase (CelA)- and *Sacchromycopsis fibuligera* β-glucosidase (BGL1)-secreting or -displaying strains. Engineering the targeted components in the ER to Golgi vesicle trafficking, including Sec12p, Sec13p, Erv25p and Bos1p, enhanced the extracellular activity of CelA. However, only Sec13p over-expression increased BGL1 secretion. By contrast, over-expression of the components in the Golgi to plasma membrane vesicle trafficking, including Sso1p, Snc2p, Sec1p, Exo70p, Ypt32p and Sec4p, showed better performance in increasing BGL1 secretion compared to CelA secretion, and the over-expression of these components all increased BGL1 extracellular activity. These results revealed that various cellulases showed different limitations in protein transport, and engineering vesicle trafficking has protein-specific effects. Importantly, we found that engineering the above vesicle trafficking components, particularly from the ER to the Golgi, also improved the display efficiency of CelA and BGL1 when a-agglutinin was used as surface display system. Further analyses illustrated that the display efficiency of a-agglutinin was increased by engineering vesicle trafficking, and the trend was consistent with displayed CelA and BGL1. These results indicated that fusion with a-agglutinin may affect the proteins’ properties and alter the rate-limiting step in the vesicle trafficking.

**Conclusions:**

We have demonstrated, for the first time, engineering vesicle trafficking from the ER to the Golgi and from the Golgi to the plasma membrane can enhance the protein display efficiency. We also found that different heterologous proteins had specific limitations in vesicle trafficking pathway and that engineering the vesicle trafficking resulted in a protein-specific effect. These results provide a new strategy to improve the extracellular secretion and surface display of cellulases in *S. cerevisiae*.

**Electronic supplementary material:**

The online version of this article (doi:10.1186/s13068-017-0738-8) contains supplementary material, which is available to authorized users.

## Background

*Saccharomyces cerevisiae* has been extensively used as a microbial cell factory for producing recombinant proteins [1–4]. As a traditional ethanol producer, *S. cerevisiae* is also an ideal candidate for a consolidated bioprocess (CBP) for cellulosic ethanol production [5, 6]. *S. cerevisiae* lacks the essential cellulases to degrade cellulose; therefore, the construction of a recombinant yeast that is capable of producing heterologous cellulases is critical for CBP. Heterologous cellulases are often secreted extracellularly or displayed on the cell surface to accomplish cellulosic ethanol production [7, 8]. However, the limitations in the yeast secretory pathway often result in relatively low protein production [9]. Therefore, the secretory pathway, including vesicle trafficking, was engineered to improve protein secretion. Vesicle trafficking is a complex process involved in many processes, including protein transport through the endoplasmic reticulum (ER), Golgi, and endosome and to either the cell membrane or vacuole, but the step that is the limiting factor for any defined secreted proteins remains poorly studied. In addition, the secretory pathway can mature both extracellular secretory protein and the surface-display protein, but the effect of vesicle trafficking on surface-displayed protein has not been studied extensively.

Vesicle trafficking can be divided into four essential steps: vesicle budding, delivery, tethering and fusion [10]. These steps are tightly regulated by Rabs, coats, tethering factors, soluble N-ethylmaleimide-sensitive factor (NSF) attachment receptor proteins (SNAREs) and a diversity of regulators. Rabs are ubiquitous monomeric Ras-like GTPases that act as molecular switches [11]. These proteins cycle between GTP- and GDP-bound states, which are mediated by guanine nucleotide exchange factors (GEFs). Tethering factors containing long putative coiled-coil proteins and multi-subunit complexes are involved in vesicle target specificity [12]. The SNARE complex assembled by v-SNAREs (vesicle membrane SNAREs) or t-SNAREs (target-membrane SNAREs) is responsible for membrane fusion [13–16].

Proteins in the secretory pathway are first folded in the ER and then transported from the ER to the Golgi. In *S. cerevisiae*, over-expression of the native protein acid phosphatase Pho5p resulted in the accumulation of core-glycosylated Pho5p in the ER, indicating that one of the rate-limiting steps of the secretory pathway is protein transport from the ER to the Golgi apparatus [17]. The proteins in the Golgi are trafficked to the cell membrane, to the vacuole or back to the ER. In *S. cerevisiae*, the over-expression of SNAREs, such as Sso1p, Sso2p Snc1p, Snc2p and Sec9p effectively enhanced the secretion of heterologous proteins, such as *Bacillus* α-amylase, *Trichoderma reesei* cellobiohydrolase Cel7A, *Talaromyces emersonii* Cel7A and *Saccharomycopsis fibuligera* β-glucosidase Cel3A [18, 19]. Over-expression of Sec1p, the Sec1/Munc18 (SM) protein, facilitating vesicle fusion by the interacting with the SNARE complex, enhanced the extracellular production of the insulin precursor and α-amylase [13, 20–23]. It was also reported that over-expression of Sec4p yielded a threefold increase in the secretion of α-amylase [24]. These results revealed that engineering vesicle trafficking is a useful strategy for efficient extracellular secretion of heterologous protein.

Although engineering vesicle trafficking has been widely studied for improving extracellular secretion of heterologous proteins, their effect on the surface-displayed proteins was not reported in *S. cerevisiae* before. In general, the C-terminal glycosylphosphatidylinositol (GPI) domain of yeast cell wall proteins was fused with heterologous proteins for the surface display, and yeast a-agglutinin Aga1p–Aga2p was a frequently used GPI-anchored protein [25, 26]. The display of cellulases on yeast surface is a promising strategy for cellulosic ethanol production. Thus, many efforts have been made to improve the surface display efficiency of heterologous proteins [27–30]. Localization of GPI-anchored proteins on cell surface also requires the correct folding, modification and transport by the secretory pathway. It is reported that the deletion of *MNN2*, a mannosyltransferase involving in protein *N*-glycosylation for *N*-glycans elongation in Golgi, improved the display levels of *Aspergillus aculeatus* β-glucosidase and *T. reesei* endoglucanase II [29], demonstrating the importance of engineering secretory pathway for the surface display of heterologous proteins. Therefore, in this work, we studied the effect of engineered protein trafficking on not only extracellular secreted proteins but also surface-displayed proteins.

Cellulases, including endoglucanase (CelA) from *C. thermocellum* and β-glucosidase (BGL1) from *S. fibuligera*, were expressed as the reporter proteins in our vesicle trafficking engineered strains. The vesicle trafficking components in ER to Golgi (Sec12p, Sec13p Erv25p and Bos1p) or Golgi to cell membrane transport (Sso1p, Snc2p, Sec1p, Exo70p, Sec4p and Ypt32p) were engineered. Sec12p, the GEF protein required for the initiation of COPII vesicle formation, enhances the membrane association of the GTPase Sar1p to promote the formation of vesicles [31]. Vesicle coats play essential roles in budding from a donor membrane and specificity for vesicle targeting. Sec13p, which is a subunit of the COPII vesicle coat, rigidifies the COPII cage and increases its membrane-bending capacity [32]. Erv25p, which is a component of COPII-coated vesicles, is responsible for collecting specific cargo, such as the secreted protein invertase but not the α factor [33]. Bos1p is an essential v-SNARE involved in ER–Golgi membrane fusion [34]. The t-SNARE Sso1p and v-SNARE Snc2p are required for the fusion of Golgi-derived vesicles with the plasma membrane and it has been reported that their over-expression enhanced heterologous protein secretion. The SM protein Sec1p interacts with the SNARE complex to stimulate vesicle fusion with the plasma membrane [35]. The GTPases Ypt32p and Sec4p function as part of the Rab cascade, in which Ypt32p recruits the GEF Sec2p to activate Sec4p and regulate the trafficking of polarized vesicles to plasma membrane through their effectors [1, 36]. Exo70p is involved in the localization of the exocyst to the plasma membrane [37].

We found that the ER to Golgi vesicle trafficking components Sec12p, Sec13p, Erv25p and Bos1p can enhance the extracellular secretion of CelA, whereas the Golgi to plasma membrane vesicle trafficking components Sso1p, Snc2p, Sec1p, Exo70p, Ypt32p and Sec4p showed better performance in increasing BGL1 extracellular secretion. Importantly, we reported the positive effect of engineering vesicle trafficking on the surface–displayed proteins for the first time. The modifications of both the ER to Golgi and the Golgi to plasma membrane vesicle trafficking increased the surface display efficiency of CelA and BGL1 through yeast a-agglutinin Aga1p–Aga2p.

## Methods

### Strains, media and growth conditions

The recombinant yeast plasmids and strains used in this study were listed in Additional file 1: Table S1. *S. cerevisiae* strain CEN.PK102-5B [38] was used as the background strain and cultivated at 30 °C in YPD medium (10 g/L yeast extract, 20 g/L peptone, 20 g/L glucose) on a rotary shaker (200 rpm) in 100 mL flasks with a 40-mL working volume. All recombinant strains were grown in SC-2xSCAA without Leucine or Leucine and Histone for heterologous protein secretion [39]. SC-2xSCAA was composed of 20 g/L glucose, 6.9 g/L yeast nitrogen base minus amino acids, 2 g/L KH_2_PO_4_ (pH 6 by KOH), 190 mg/L arginine, 108 mg/L methionine, 52 mg/L tyrosine, 290 mg/L isoleucine, 440 mg/L lysine, 200 mg/L phenylalanine, 1260 mg/L glutamic acid, 400 mg/L aspartic acid, 380 mg/L valine, 220 mg/L threonine, 130 mg/L glycine, 400 mg/L leucine, 40 mg/L tryptophan and 140 mg/L histone. *Escherichia coli* Trans 5α was used to construct the plasmids and the strains were cultivated in Luria Bertani (LB, 5 g/L yeast extract, 10 g/L peptone and 10 g/L NaCl) medium with 100 μg/mL ampicillin at 37 °C.

### Plasmid and strain construction

The primers used for PCR are shown in Additional file 1: Table S2. All recombinant plasmids were constructed using the Gibson method [40]. *AGA1* was amplified from the CEN.PK102-5B genomic DNA and ligated into pJFE3 between the *TEF1* promoter and *PGK1* terminator [41]. The recombinant plasmid was named pJFE3-AGA1. The *BGL1* fragments [42] fused with the flag tag and *AGA2* were inserted in the pIYC04 plasmid under the control of *TEF1* promoter and *ADH1* terminator. *TEF1*p-*AGA2*-*BGL1*-*ADH1*t was amplified and cloned into pJFE3-AGA1 to construct the A12-BGL plasmid. The *CelA* fragments fused with the myc tag [42] and *AGA2* were inserted in the pIYC04 plasmid under the control of the *PGK1* promoter and *CYC1* terminator. *PGK1*p-*AGA2*-*CelA*-*CYC1*t was amplified and ligated to pJFE3-AGA1 to construct the A12-CEL plasmid. The A12-BGL and A12-CEL plasmids were transformed into CEN.PK102-5B, and the resulting strains were named A12THB0 and A12THC0. The yeast 2 μ plasmid pYX242WS [43], which contains the *LEU2* gene as selecting marker, was used to over-express the genes involved in vesicle trafficking. The *SEC12*, *SEC13*, *ERV25*, *BOS1*, *SSO1*, *SNC2*, *SEC1*, *EXO70*, *YPT32* and *SEC4* fragments were amplified from the CEN.PK102-5B genomic DNA, inserted into the pYX242WS plasmid under the control of *TEF1* promoter and *polyA* terminator and transformed into THB0, THC0, A12THB0 and A12THC0, respectively.

### Enzymatic assays

Endoglucanase activity was measured as previously described. The supernatant was mixed with 50 mM citrate buffer (pH 4.8) and 1% sodium carboxymethylcellulose (CMC) (Sigma, USA) and incubated at 50 °C for 30 min to quantify the extracellular CelA activity. The cells were collected and washed twice with 50 mM citrate buffer to quantify the cell CelA activity. The cells were suspended in 50 mM citrate buffer and incubated in 50 mM citrate buffer with 1% CMC. The reducing sugars contents were estimated at 540 nm after boiling with the dinitrosalicylate (DNS) reagent for 10 min. One unit of enzyme activity was defined as the amount of enzyme required to release 1 μmol of reducing sugars per minute under the assay conditions.

β-Glucosidase activity was measured using the substrate *p*-nitrophenyl-β-d-glucopyranoside (*p*NPG) (Sigma, USA) [44]. The supernatant was collected and incubated in 50 mM citrate buffer (pH 5.0) with 5 mM *p*NPG for 30 min at 50 °C to measure the extracellular BGL1 activity. The cells were collected and washed two times with 50 mM citrate buffer to measure the cell activity. The cells were suspended in 50 mM citrate buffer and incubated in 50 mM citrate buffer with 5 mM *p*NPG for 30 min at 50 °C. The reaction was stopped by adding of 10% sodium carbonate, and the *p*-nitrophenol (*p*NP) released from *p*NPG was detected at 405 nm. One unit of enzyme activity was defined as the amount of enzyme required to release 1 μmol of *p*NP per minute under the assay conditions.

Invertase activity was measured as previously described [18]. The cells were grown in SCAA medium containing 2% sucrose to induce of invertase expression. The amount of glucose released from sucrose by invertase was determined using the d-glucose (GOPOD) kit (Megazyme K-CERA, Wicklow, Ireland). One unit of invertase activity was defined as the amount of enzyme required to release 1 mmol of glucose per min at 30 °C.

### Immunofluorescence microscopy and flow cytometry analysis

The cells were harvested by centrifugation at 8000×*g* and washed twice with phosphate buffered saline (PBS, pH 7.0). The cells were suspended in PBS containing 1 mg/mL bovine serum albumin (BSA) and mouse monoclonal Anti-DDDDK tag (DyLight® 488) (Abcam, UK) and Anti-Myc tag (FITC) (Abcam, UK) antibodies at 1:500 dilutions to an OD_600_ of 1.0 at 25 °C for 1 h. After the reaction, the cells were pelleted and washed twice with PBS. Images were captured using immunofluorescence microscope (Olympus, Japan) and the flow cytometry analysis (FACS) was performed with FACSCanto II (BD FACSCanto II, USA).

### Real-time quantitative PCR

Recombinant strains were grown in 40 mL of SCAA media to an OD_600_ of 0.6–0.8. The cells were harvested and frozen rapidly in liquid nitrogen. The RNA was extracted using UINQ-10 spin column RNA purification kits (BBI), according to the manufacturer’s instruction. The cDNAs were synthesized using the PrimeScript RT-PCR Kit (Takara, Japan). The SYBR Green Master Mix Kit (Roche Molecular Biochemicals, Germany) was used for the real-time quantitative PCR.

## Results

### The effect of engineering vesicle trafficking from the ER to the Golgi on heterologous protein secretion

**Fig. 1:**
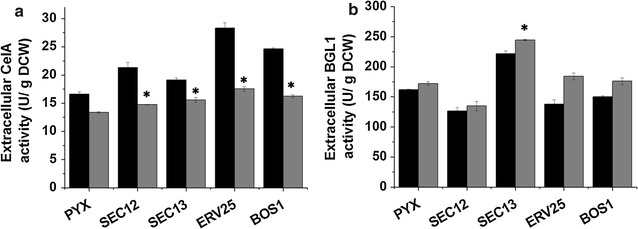
The effect of engineering the ER to Golgi vesicle trafficking on the extracellular activity of cellulases. **a** The extracellular activity of CelA. **b** The extracellular activity of BGL1. *PYX* strain expressing the empty plasmid that was used as control. The *black bars* represent 36 h, the *gray bars* represent 72 h. The data are presented as the means ± standard errors from two independent experiments. *p value of the marked sample vs. control (PYX) <0.05

Four genes, *SEC12*, *SEC13*, *ERV25* and *BOS1*, which are involved in vesicle trafficking from the ER to the Golgi were over-expressed in CelA- and BGL1-expressing strains (Additional file 1: Figure S1A), and their over-expression did not up-regulate the transcription of the heterologous cellulase genes (Additional file 1: Figures S1B, C). The extracellular cell-specific activities (U/g dry cell weight, U/g DCW) of CelA and BGL1 were measured and the results were shown in Fig. 1a. Compared with the control strain PYX, the over-expression of *SEC12*, *SEC13*, *ERV25* and *BOS1* yielded increases of 11, 17, 31 and 22% in the extracellular secretion of CelA at 72 h, respectively, indicating that engineering vesicle trafficking components from the ER to the Golgi can effectively increase CelA secretion. However, most of the ER to Golgi vesicle trafficking engineering did not show obvious effects on BGL1 secretion, and only the Sec13p over-expressing strain resulted in a 40% increase in the extracellular activity of BGL1 (Fig. 1b). The other components did not have a positive effect on extracellular BGL1 activity. Trends in the extracellular activities of CelA and BGL1 from the Sec12p, Sec13p, Erv25p and Bos1p expressing strains at 36 h were similar to the trends at 72 h. These results illustrated that engineering vesicle trafficking from the ER to the Golgi had a more obvious impact on the extracellular activity of CelA.

### The effect of engineering vesicle trafficking from the Golgi to the plasma membrane on heterologous protein secretion

**Fig. 2:**
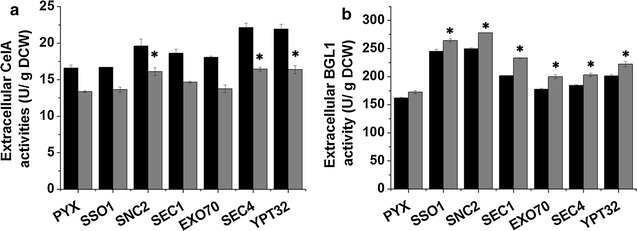
The effect of engineering the Golgi to plasma membrane vesicle trafficking on the extracellular activity of cellulases. **a** The extracellular activity of CelA. **b** The extracellular activity of BGL1. *PYX* strain expressing the empty plasmid that was used as control. The *black bars* represent 36 h, the *gray bars* represent 72 h. The data are presented as the means ± standard errors from two independent experiments. *p value of the marked sample vs. control (PYX) <0.05

The effect of engineering vesicle trafficking from the Golgi to the plasma membrane on heterologous protein secretion was also studied, and the key components Sso1p, Snc2p, Sec1p, Exo70p, Sec4p and Ypt32p were over-expressed (Additional file 1: Figure S1A). These modifications did not up-regulate the transcription of the heterologous genes (Additional file 1: Figures S1B, C). Compared with the control strain (Fig. 2a), over-expression of Snc2p, Sec4p and Ypt32p increased the extracellular activity of CelA by 20, 22 and 23%, respectively, whereas over-expression of Sso1p, Sec1p and Exo70p did not increase the extracellular activity of CelA. By contrast, all these modifications of vesicle trafficking from the Golgi to the plasma membrane improved the secretion of BGL1 (Fig. 2b). Over-expression of the SNAREs Sso1p and Snc2p achieved the greatest increases in BGL1 secretion of 53 and 61%, respectively. In addition, over-expression of Sec1p, Exo70p, Sec4p and Ypt32p markedly increased the extracellular activity of BGL1. These results showed that the ER to Golgi transport is more beneficial for CelA secretion but that engineering the Golgi to plasma membrane transport clearly improved BGL1 secretion.

### Yeast surface display of CelA and BGL1 through a-agglutinin

**Fig. 3:**
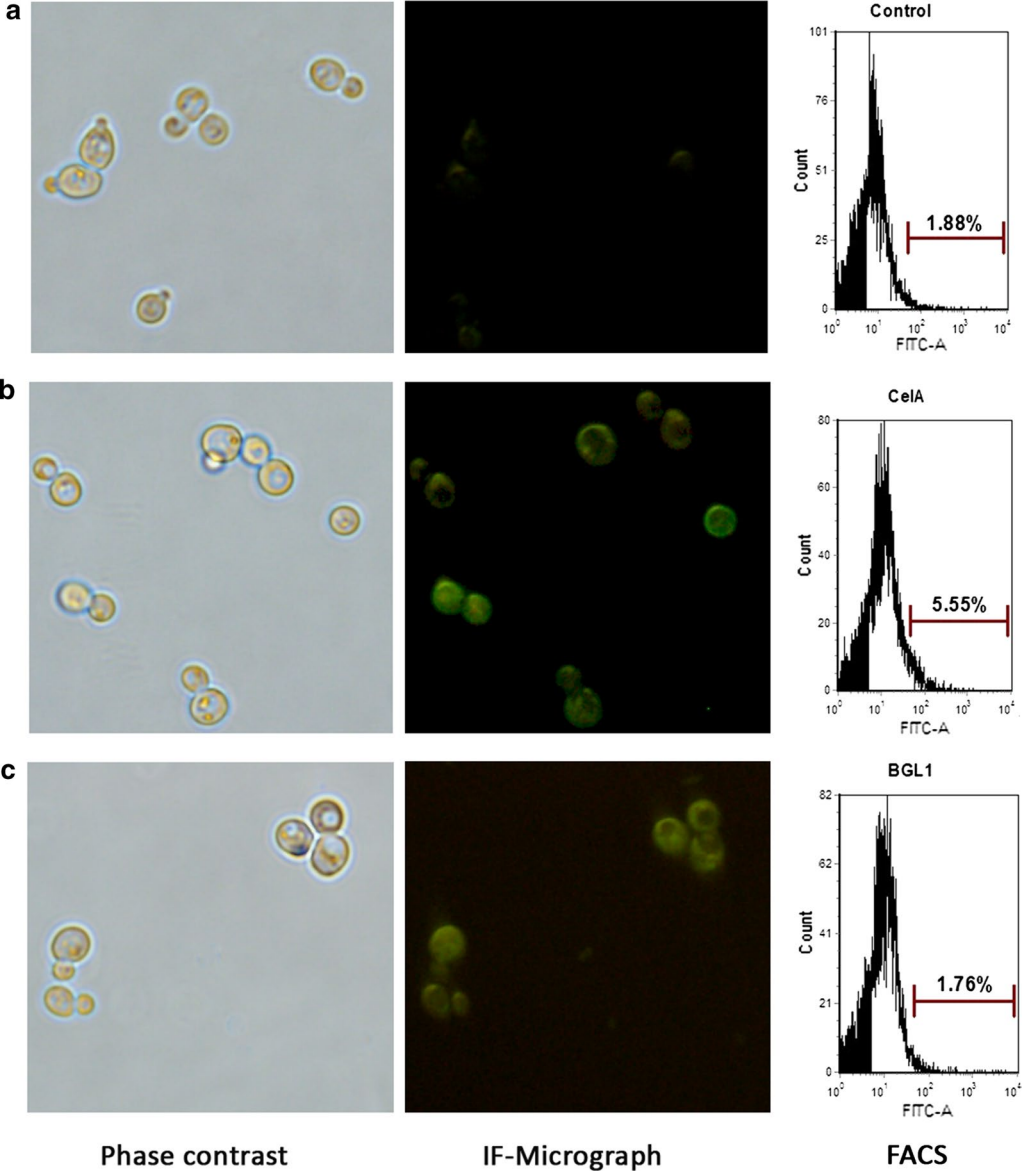
Immunofluorescence micrographs and FACS analysis of the surface display of CelA and BGL1. **a** Immunofluorescence micrographs and FACS analysis of the control strain with empty plasmid. **b** Immunofluorescence micrographs and FACS analysis of the displayed BGL1. **c** Immunofluorescence micrographs and FACS analysis of the displayed CelA. The results are representative of two independent experiments using two individual clones

Currently, the cell wall localization of heterologous proteins is widely used for biocatalysts [25]. The display of cellulases on *S. cerevisiae* is also an important strategy for CBP construction [45]. Thus, it is necessary to increase the surface display efficiency of heterologous cellulases to develop a CBP. Yeast a-agglutinin Aga1p–Aga2p was frequently used as an anchor protein for the cell surface display of heterologous proteins [25]. CelA and BGL1 were fused with Aga2p and co-expressed with Aga1p for cell wall localization. The expression of CelA and BGL1 on the yeast cell surface was confirmed with immunofluorescence microscopy and flow cytometry analyses (FACS). As shown in Fig. 3 (fluorescence photos), recombinant strains displaying CelA (Fig. 3b) and BGL1 (Fig. 3c) showed clear fluorescence signals, whereas the control strain with the empty plasmid was not immunostained (Fig. 3a). The FACS analysis of CelA showed that 5.55% of the population was positively stained. However, the FACS analysis did not detect the stained BGL1-displaying strain. Further analysis determined that the percentage of cell activity [cell activity/(cell activity + extracellular activity)] in the BGL1-displaying strain (95–98%) was higher than the secreted strain (70–75%) (Additional file 1: Figure S2), indicating that BGL1 was also successfully displayed on the cell surface, but the display efficiency was at a low level that could not be detected using FACS.

### Engineering vesicle trafficking from the ER to the Golgi improved the surface display efficiency of heterologous proteins

**Fig. 4:**
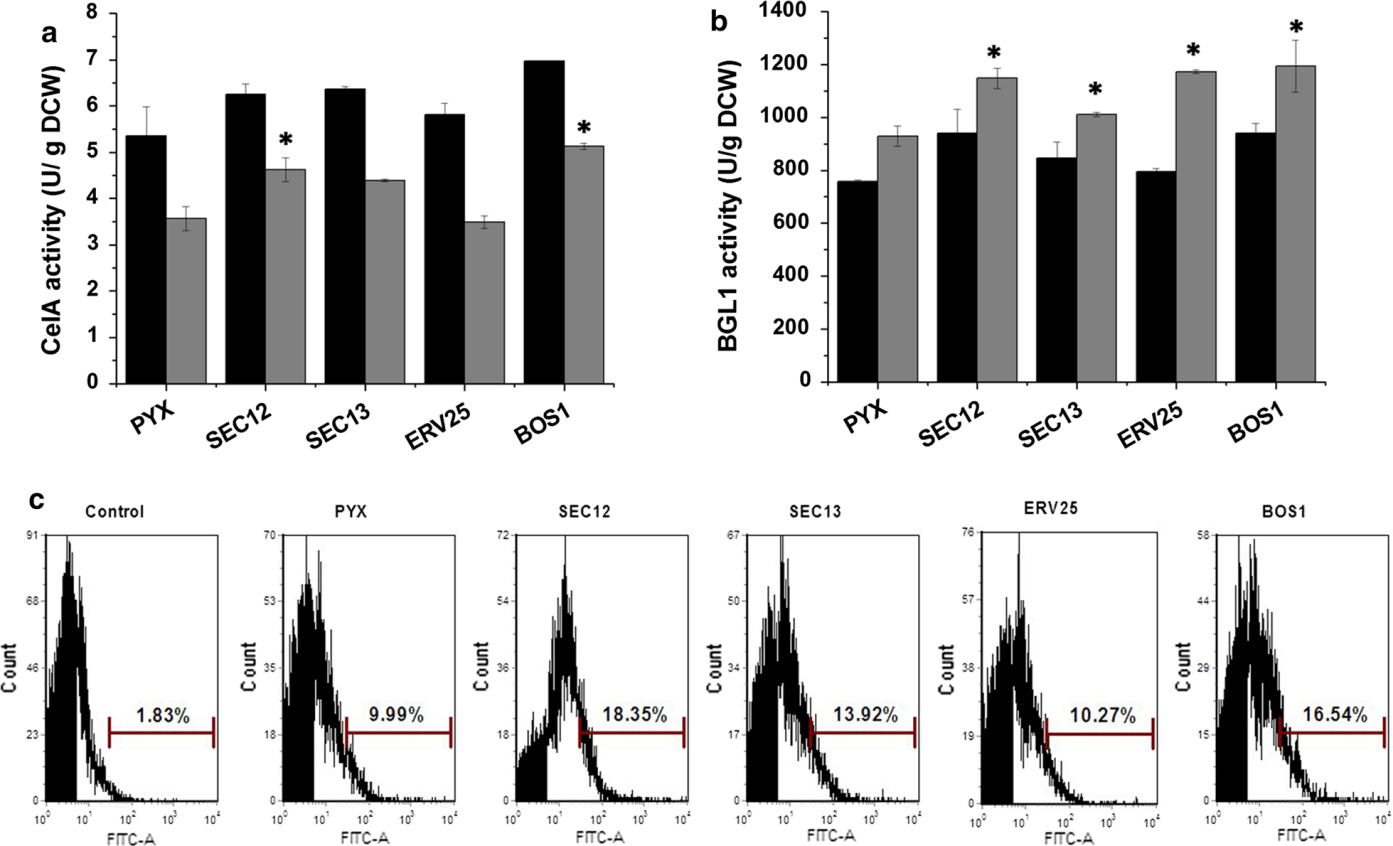
Engineering the ER to Golgi vesicle trafficking improved the display efficiency of heterologous proteins. **a** The cell activity of CelA in the ER to Golgi vesicle trafficking engineered strains. **b** The cell activity of BGL1 in the ER to the Golgi vesicle trafficking engineered strains. **c** FACS analysis of CelA display efficiency in the ER to Golgi vesicle trafficking engineered strains. Control: the strains without expressing heterologous protein was used as the negative control. The *black bars* represent 36 h, the *gray bars* represent 72 h. The data are presented as the means ± standard errors from two independent experiments. *p value of the marked sample vs. control (PYX) <0.05

Both cell activities and FACS were used to analyze the effect of engineering vesicle trafficking from the ER to the Golgi on the surface display level of CelA and BGL1. As shown in Fig. 4a, the cell activity of CelA was increased by over-expression of Sec12p, Sec13p and Bos1p, and among them, the highest improvement achieved 71% in Bos1p expressing strain, compared with the control strain. Consistent with the enzyme activity, the FACS analysis also showed the increased surface display levels in Sec12p, Sec13p and Bos1p expressing strains (Fig. 4c). Among the strains, the over-expression of Sec12p and Bos1p increased the percentage of immunostained cells by 83 and 65%, respectively. In addition, the cell activities of surface-displayed BGL1 were improved in the four engineered strains (Fig. 4b). Over-expression of Sec12p, Sec13p, Erv25p and Bos1p resulted in 24, 9, 26 and 29% increases in BGL1 activity, respectively, compared with the control strain. However, the FACS data did not show any differences (data were not shown). These results revealed that enhancing protein transport from the ER to the Golgi was an effective strategy for the cell surface display of both CelA and BGL1 through a-agglutinin Aga1p–Aga2p. The effects of these strains on displayed CelA were consistent with secreted CelA, but the effects on displayed BGL1 were more obvious than secreted BGL1.

### Engineering vesicle trafficking from the Golgi to the plasma membrane improved the surface display efficiency of heterologous proteins

**Fig. 5:**
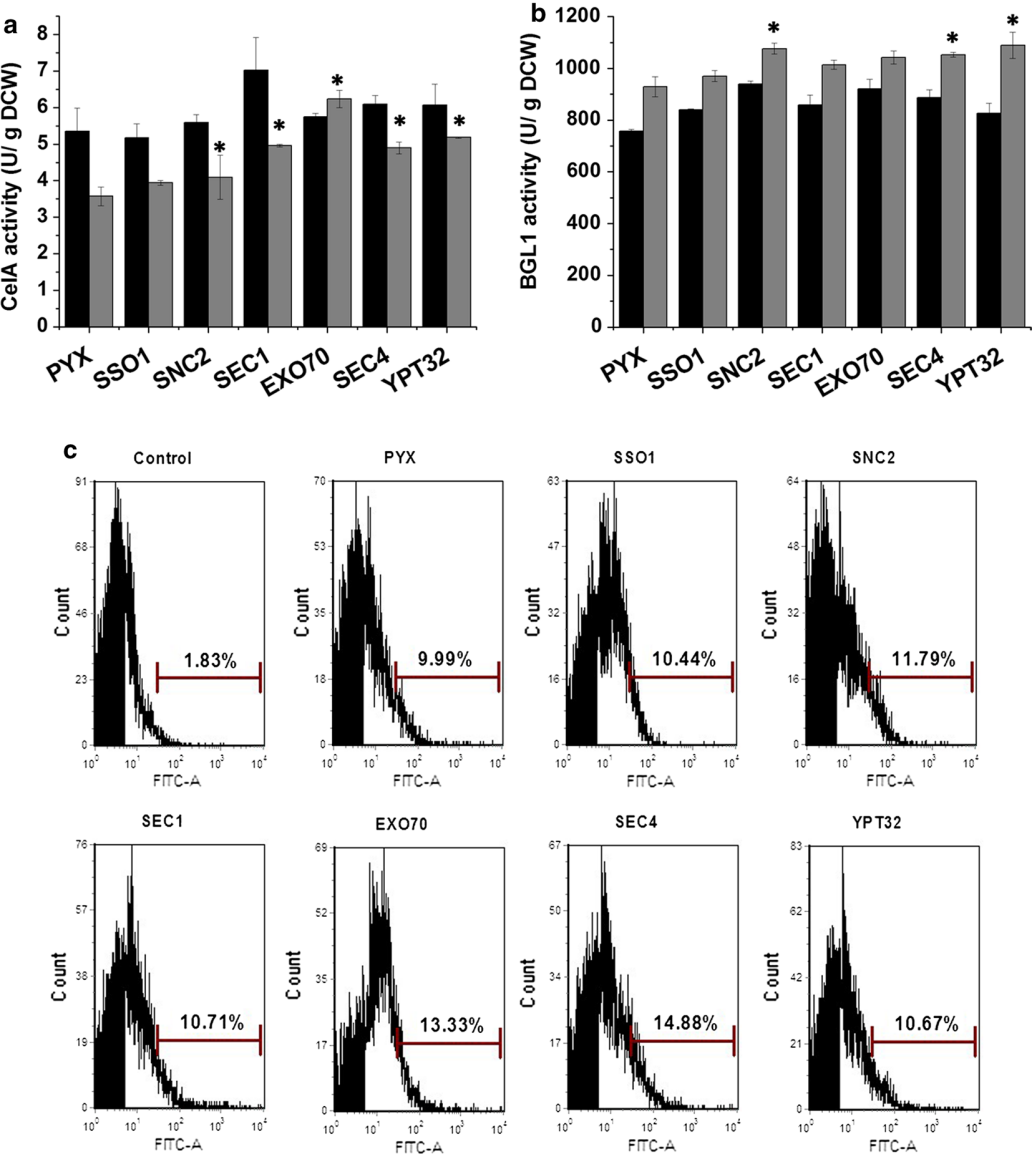
Engineering the Golgi to plasma membrane vesicle trafficking increased the display efficiency of heterologous proteins. **a** The cell activity of CelA in the Golgi to membrane vesicle trafficking engineering strains. **b** The cell activity of CelA in the Golgi to membrane vesicle trafficking engineering strains. **c** FACS analysis of CelA display efficiency in the strains expressing the Golgi to plasma membrane vesicle trafficking components. The *black bars* represent 36 h, the *gray bars* represent 72 h. The data are presented as the means ± standard errors from two independent experiments. *p value of the marked sample vs. control (PYX) <0.05

The effect of engineering vesicle transport from the Golgi to the plasma membrane on the surface display efficiency was also analyzed and the results were shown in Fig. 5. Over-expression of Snc2p, Sec1p, Exo70p, Sec4p and Ypt32p improved the cell activity of CelA. The trends of the FACS analysis were similar to the trends in CelA activity (Fig. 5c), and over-expression of Exo70p yielded a maximal 33% increase in the percentage of immunostained cells. However, Sso1p did not enhance the display efficiency of CelA. Over-expression of Snc2p, Sec4p and Ypt32p improved the cell activity of BGL1 by 16, 13 and 17%, respectively. Over-expression of Sec1p and Exo70p also slightly improved the cell activity of BGL1. The results indicated that over-expression of the key vesicle trafficking components from the Golgi to the plasma membrane can also improve the surface display efficiency of both CelA and BGL1 through Aga1p–Aga2p, and the impacts of these modifications on displayed CelA and BGL1 were similar to those observed for secreted CelA and BGL1.

**Fig. 6:**
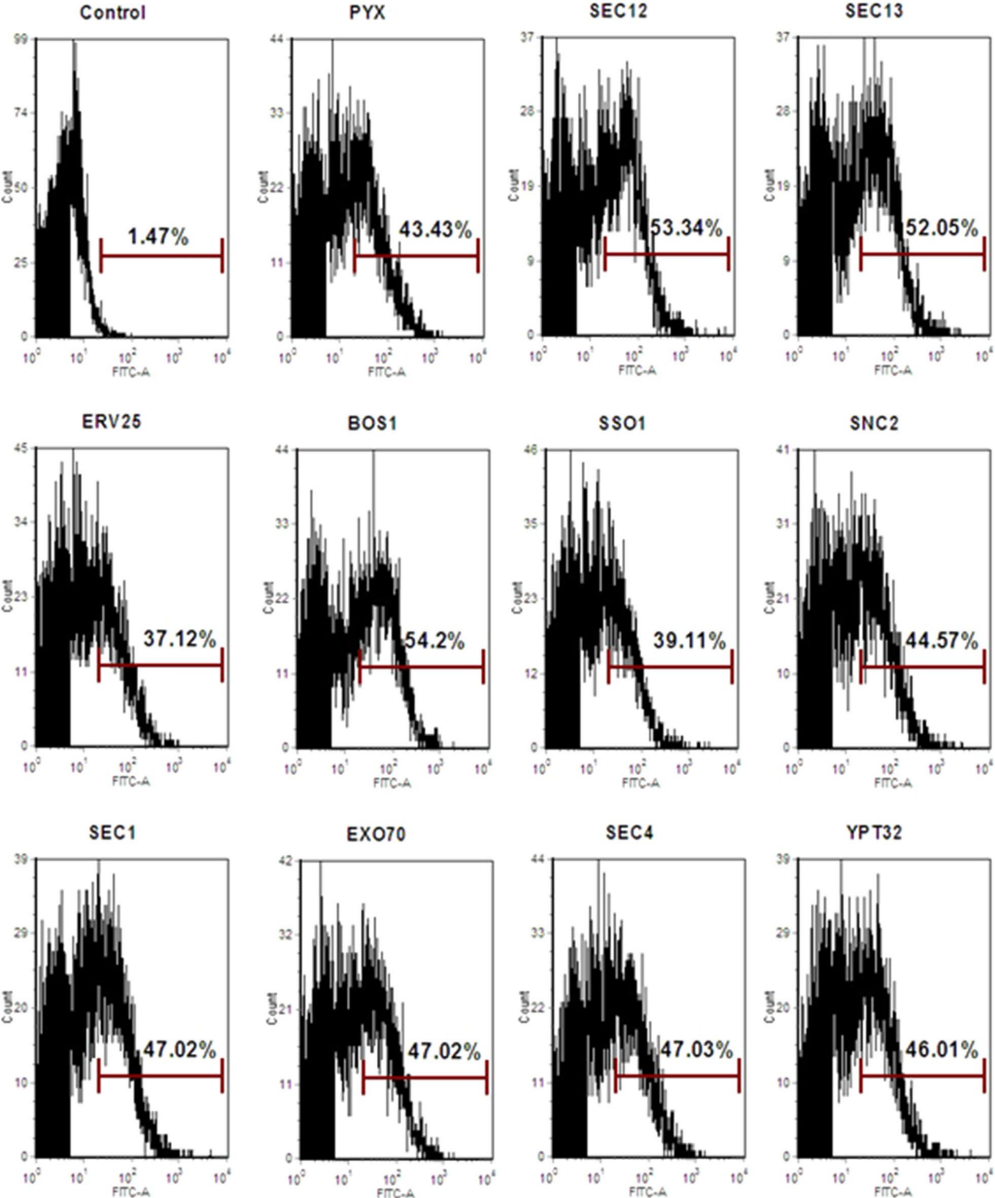
The effect of engineering vesicle trafficking on the display efficiency of a-agglutinin. The results are representative of two independent experiments using two individual clones

We also analyzed the cell surface display efficiency of yeast a-agglutinin Aga1p–Aga2p in the vesicle trafficking engineered strains to investigate whether engineering protein transport affects the display of a-agglutinin (Fig. 6). The results showed that engineering of most of the vesicle trafficking components increased the percentage of immunostained cells and that the effects of the modifications of vesicle trafficking from the ER to the Golgi were more significant than the effects of modifications of vesicle trafficking from the Golgi to the plasma membrane. This trend was similar to the trends of displayed CelA and BGL1.

### Improvements in endogenous invertase secretion by modifying vesicle trafficking

**Fig. 7:**
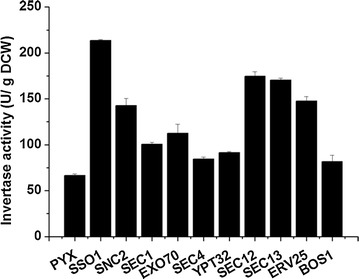
Specific activity of the endogenous invertase in the vesicle trafficking engineered strains. The cells were grown on 2% sucrose and collected at 12 h. The data are presented as the means ± standard errors from two independent experiments

The secretion of the endogenous invertase was also investigated in these vesicle trafficking engineered strains. As shown in Fig. 7, the invertase activity was significantly improved in both the ER to Golgi and the Golgi to plasma membrane vesicle trafficking engineered strains. In particular, over-expression of Sso1p resulted in a 2.2-fold increase in invertase secretion, and over-expression of Snc2p, Sec12p, Sec13p and Erv25 increased invertase activity by 1.14-, 1.62-, 1.56- and 1.21-fold, respectively. These results indicated that optimizing the secretory pathway by strengthening vesicle trafficking from the ER to the Golgi and from the Golgi to the plasma membrane was beneficial for both heterologous and endogenous protein secretion.

## Discussion

Cellulase expression in *S. cerevisiae* can enhance cellulose hydrolysis and ethanol production by simultaneous saccharification and fermentation (SSF) and CBP [46, 47]. Currently, the production of cellulases generally follows two strategies: either the extracellular secretion of the enzymes or displaying the enzymes on the cell surface. Increasing cellulase activity can further improve the production of cellulosic ethanol [48, 49]. Engineering vesicle trafficking from the ER to the Golgi and from the Golgi to the plasma membrane can improve the extracellular secretion of cellulases [50]. However, the step that is the limiting factor for any defined secreted protein remains poorly studied, as is the effect of engineering vesicle trafficking on surface-displayed protein. Thus in this study, we investigated the effects of multiple components involved vesicle budding, tethering and fusion on the efficiencies of both extracellular secretion and surface display of cellulases.

**Fig. 8:**
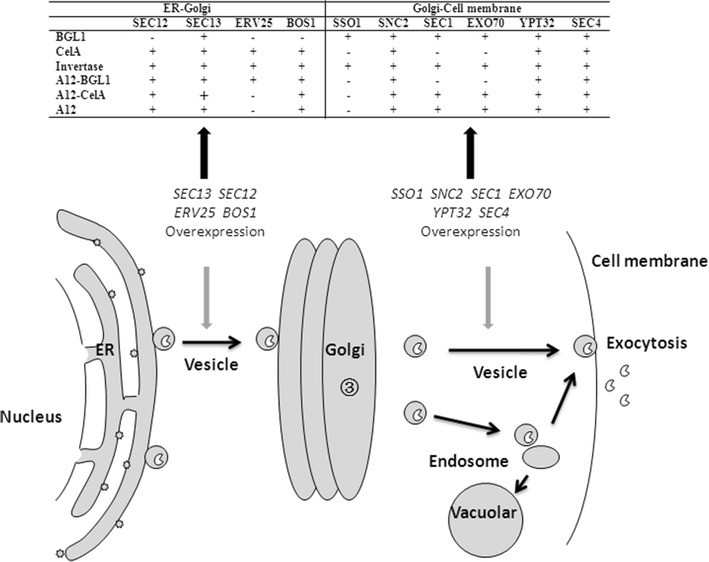
Engineering vesicle trafficking process improved the secretion of BGL1, CelA and invertase and the surface display efficiency of BGL1, CelA and a-agglutinin. *Plus* represents over-expressing the defined genes had the positive effect to protein secretion or display; - represents over-expressing the defined genes had no positive effect to protein secretion or display

BGL1 contains six *N*-glycosylation sites (predicted by NetNGlyc 1.0 Server) and four potential disulfide bonds (predicted by DiANNA 1.1 web server) with a theoretical molecular weight of 96.2 KD. CelA contains two *N*-glycosylation sites and no disulfide bond with a theoretical molecular weight of 52 KD. Previously, it was reported the large surface proteins of the hepatitis B virus (HBV) with a large size of preS1 domain were accumulated in ER and provoked a proliferation, but not the major and middle surface proteins [51]. *T. reesei* endoglucanase I (EG I) was an ER-accumulated heterologous protein in *S. cerevisiae* and the over-expression of the Sso2p involved in Golgi to plasma membrane vesicle trafficking did not increase its extracellular activity [18]. *Pyrococcus furiosus* β-glucosidase with no disulfide bond in *S. cerevisiae* resulted in the accumulation of mostly inactive β-glucosidase in the ER, and over-expression of Pdi1p, a disulfide isomerase responsible for the correct formation of disulfide bonds, increased its extracellular secretion significantly [52]. *P. furiosus* β-glucosidase with no disulfide bond in *S. cerevisiae* resulted in the accumulation of mostly inactive β-glucosidase in the ER and over-expression of Pdi1p, a disulfide isomerase responsible for the correct formation of disulfide bonds, increased its extracellular secretion significantly. These studies showed that heterologous proteins with different properties presented different restrictions in the vesicle trafficking process and may result in protein-specific effects of engineering vesicle trafficking from the ER to the Golgi and from the Golgi to the plasma membrane. Our results showed that engineering the vesicle trafficking components from Golgi to plasma membrane can improve the secretion of *C. thermocellum* endoglucanase CelA, *S. fibuligera* β-glucosidase BGL1 and the endogenous invertase, while over-expression the protein transport components from the ER to the Golgi had more obvious effect on the secretion of CelA and invertase (Fig. 8). We found that the plasmid copy number of CelA was higher than BGL1 (Additional file 1: Figure S3), indicating that CelA had higher expression levels than BGL1. High CelA expression may result in the folding capacity saturation and protein accumulation in the ER. Strengthening the vesicle trafficking pathway out of ER may relieve the protein accumulation and contribute to the extracellular secretion of CelA. Previous studies illustrated that over-expression of the SNAREs Sso1p, Snc1p and Sec9p involved in the Golgi to plasma membrane vesicle trafficking, but not the ER to Golgi vesicle trafficking SNAREs Bos1p, Bet1p and Sec22p, increased the extracellular secretion of *S. fibuligera* BGL1 [50], which was consistent with our results. However, we still cannot give a direct conclusion on the relationship between the protein properties and the restriction step in the vesicle trafficking process based on our data.

Yeast a-agglutinin Aga1p–Aga2p is the most widely used surface anchor protein for displaying heterologous proteins [53, 54]. The fusion of a-agglutinin​, as a glycosylphosphatidylinositol (GPI)-anchored protein, turns the extracellular recombinant proteins into cell wall proteins containing the GPI domain. Thus, the impacts of engineering on the displayed CelA and BGL1 were similar to a-agglutinin, but were not completely consistent with the secreted proteins. GPI-anchored proteins constitute a special category of cargo protein and require the defined cargo receptor/adaptor which is different from the secreted proteins [55]. In the vesicle trafficking from the ER to the Golgi, GPI-anchored proteins were recognized and concentrated by a transmembrane cargo receptor/adaptor and the p24 complex for the ER exit [56, 57]. In the protein transport from the Golgi to the cell membrane, GPI-anchored proteins and secreted proteins were also transported by different vesicles [58]. The major vesicle population contains cell wall GPI-anchored protein Bg12p and plasma membrane protein Pma1p, while another population contains the secreted enzymes, such as invertase and acid phosphatase [59]. Some vesicle trafficking components involved in GPI-anchored protein transport are shared with the secreted protein transport components, while a small part of components are different [59, 60]. We speculated that fusion of CelA and BGL1 with Aga2p and co-expression with Aga1p can form recombinant proteins containing GPI domain, which makes the properties of recombinant CelA and BGL1 to be GPI-anchored proteins (Fig. 8). In addition, we found although fold change varies between FACS data and activity data, the trends of the FACS data were similar to the enzyme activities. The activities represent active enzymes, while the FACS data showed the display of total enzymes which may contain a fraction of inactive enzymes. The inactive enzyme can result from protein mis-folding. This may be the reason for differences between the values of activity and FACS.

For efficient surface display of heterologous proteins, many efforts have been made. The anchor domain of various cell wall proteins was compared, and it is reported that the anchor domain of Sed1p improved the activity of β-glucosidase from *Aspergillus aculeatus* and endoglucanase II from *T. reesei* on cell surface significantly, compared to α-agglutinin Agα1p [27, 28]. The optimization of promoter and signal peptide of Sed1p also significantly increased the display efficiency of both *A. aculeatus* β-glucosidase and *T. reesei* endoglucanase II [27, 60]. In addition, optimization of linker between heterologous protein and anchor domain has been performed to elevate display efficiency of heterologous proteins [61]. These above strategies were mainly engineering the expression vector system and the protein anchoring system for increasing the transcription and translation level. However, many proteins are still secreted at relatively low levels even though the transcription or translation of heterologous proteins is optimized [62]. This indicates that post-translational modification in secretory pathway was one of key potential limitations. Our results showed that engineering vesicle trafficking improved the surface display efficiency of heterologous proteins, which also demonstrated engineering secretory pathway is a promising strategies for efficient surface display of heterologous proteins. In addition, the combinational overexpression of our selected components together with these strategies may further improve the display and secretion of heterologous proteins.

## Conclusions

In this study, we have shown that engineering vesicle trafficking from the ER to the Golgi and from the Golgi to the plasma membrane not only increased cellulase secretion, but also, for the first time, improved the production of displayed cellulases. Engineering protein transport from the ER to the Golgi and from the Golgi to the plasma membrane by modifying the proteins involved in vesicle budding, tethering and fusion had a protein-specific effect, and the fusion of a-agglutinin for cell surface display may change the proteins’ properties, thereby altering the rate-limiting step in the secretory pathway. Our research showed that modifying the vesicle trafficking process is a promising approach for enhancing the extracellular secretion and surface display of heterologous proteins.

## Additional file

**Additional file 1: Figure S1.** Transcription level of the vesicle trafficking components and heterologous proteins. **Figure S2.** The percentage of cell activity of BGL1 (cell activity/ (cell activity + extracellular activity)) in the surface-displayed BGL1 and secreted BGL1 strains. **Figure S3.** The copy number of plasmids expressing heterologous cellulase genes and vesicle trafficking genes, respectively. **Table S1.** Strains and plasmids used in this study. **Table S2.** The primers used in this study.

## Abbreviations

CBP: consolidated bioprocess
ER: endoplasmic reticulum
BGL1: β-glucosidase
CelA: endoglucanase
SNAREs: soluble NSF [N-ethylmaleimide-sensitive factor] attachment receptor proteins
v-SNAREs: vesicle membrane SNAREs
t-SNAREs: target-membrane SNAREs
Rabs: ras-like GTPases
GEFs: guanine nucleotide exchange factors
SM: Sec1/Munc18
LB: Luria–Bertani
CMC: carboxymethylcellulose
DNS: dinitrosalicylate
*p*NPG: *p*-nitrophenyl-β-d-glucopyranoside
FACS: flow cytometry analyses
DCW: dry cell weight
COP II: coat protein complex II
SSF: simultaneous saccharification and fermentation
