# Supplementary material for: Engineering vesicle trafficking improves the extracellular activity and surface display efficiency of cellulases in Saccharomyces cerevisiae
Source: Biotechnol Biofuels. 2017 Feb 27;10:53. doi: 10.1186/s13068-017-0738-8 (PMC5327580; doi:10.1186/s13068-017-0738-8)
Supplement: Supplementary file 1 — Additional file 1: Figure S1. Transcription level of the vesicle trafficking components and heterologous proteins. Figure S2. The percentage of cell activity of BGL1 (cell activity/ (cell activity + extracellular activity)) in the surface-displayed BGL1 and secreted BGL1 strains. Figure S3. The copy number of plasmids expressing heterologous cellulase genes and vesicle trafficking genes, respectively. Table S1. Strains and plasmids used in this study. Table S2. The primers used in this study. [file 13068_2017_738_MOESM1_ESM.docx]

**Engineering vesicle trafficking improves the extracellular activity and surface display efficiency of cellulases in *Saccharomyces cerevisiae***

Hongting Tang^1^, Meihui Song^1^, Yao He^1^, Jiajing Wang^1^, Shenghuan Wang^1^, Yu Shen^1^, Jin Hou^1^*, Xiaoming Bao^1,2^

^1^State Key Laboratory of Microbial Technology, The Collage of Life Science, Shandong University, Jinan, 250100, China

^2^Shandong Provincial Key Laboratory of Microbial Engineering, Qi Lu University of Technology, Jinan 250353, PR China

* Corresponding author: Dr. Jin Hou, email: [houjin@sdu.edu.cn](mailto:houjin@sdu.edu.cn), State Key Laboratory of Microbial Technology, The Collage of Life Science, Shandong University, Jinan 250100, China. Tel.: +86 531 8836 5827; Fax: +86 531 8836 5826


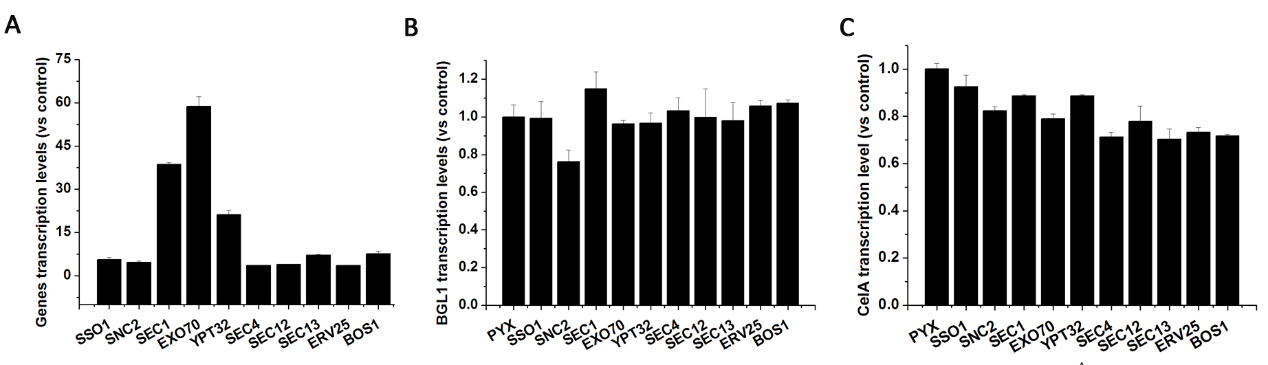


Fig. S1 Transcription level of the vesicle trafficking components and heterologous proteins. A. Transcription of the over-expressed vesicle trafficking genes. B. Transcription of *BGL1* in the vesicle trafficking engineered strains. C. Transcription of *CelA* in the vesicle trafficking engineered strains. The data are presented as the means ± standard errors from two independent experiments.


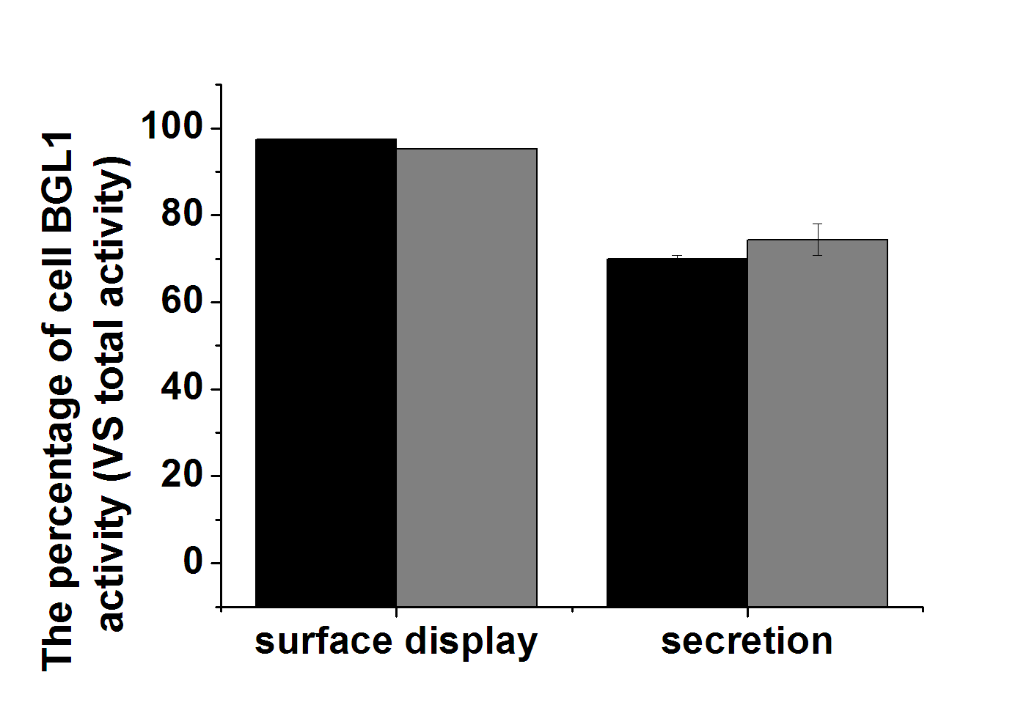


Fig. S2 The percentage of cell activity of BGL1 (cell activity/ (cell activity + extracellular activity)) in the surface-displayed BGL1 and secreted BGL1 strains. The black bars represent 36 h, the grey bars represent 72 h. The data are presented as the means ± standard errors from two independent experiments.





Fig. S3 The copy number of plasmids that expressing heterologous cellulases genes and vesicle trafficking genes, respectively. The black bars represent 36 h, the grey bars represent 72 h. The data are presented as the means ± standard errors from two independent experiments.

Table S1 Strains and plasmids used in this study.

| Strain or plasmid | Genotype | Reference |
| --- | --- | --- |
| Plasmids |  |  |
| pTH-BGL | pIYC04 with *TPI1* promoter, *PGI1* terminator and *S. fibuligera BGL1* | [47] |
| pTH-CEL | pIYC04 with *PGK1* promoter, *CYC1* terminator and *C. thermocellum CelA* | [47] |
| pJFE3 | Yeast 2μ plasmid with URA3 marker | [46] |
| A12-BGL | pJFE3 with *TEF1* promoter, *ADH1* terminator, *BGL1* fused with *AGA2* and with *TEF1* promoter, *PGK1* terminator, *AGA1* | This study |
| A12-CelA | pJFE3 with *PGK1* promoter, *CYC1* terminator, *CelA* fused with *AGA2* and with *TEF1* promoter, *PGK1* terminator, *AGA1* | This study |
| A12 | pJFE3 with *PGK1* promoter, *CYC1* terminator, *AGA2* and with *TEF1* promoter, *PGK1* terminator, *AGA1* | This study |
| pYX242WS | Yeast 2μ plasmid with LEU marker | [48] |
| SSO1 | pYX242WS with *TEF1* promoter, *PolyA* terminator and *SSO1* gene | This study |
| SNC2 | pYX242WS with *TEF1* promoter, *PolyA* terminator and *SNC2* gene | This study |
| SEC1 | pYX242WS with *TEF1* promoter, *PolyA* terminator and *SEC1* gene | This study |
| EXO70 | pYX242WS with *TEF1* promoter, *PolyA* terminator and *EXO70* gene | This study |
| YPT32 | pYX242WS with *TEF1* promoter, *PolyA* terminator and *YPT32* gene | This study |
| SEC4 | pYX242WS with *TEF1* promoter, *PolyA* terminator and *SEC4* gene | This study |
| SEC12 | pYX242WS with *TEF1* promoter, *PolyA* terminator and *SEC12* gene | This study |
| SEC13 | pYX242WS with *TEF1* promoter, *PolyA* terminator and *SEC13* gene | This study |
| ERV25 | pYX242WS with *TEF1* promoter, *PolyA* terminator and *ERV25* gene | This study |
| BOS1 | pYX242WS with *TEF1* promoter, *PolyA* terminator and *BOS1* gene | This study |
| Strains |  |  |
| CEN.PK102-5B | *MATa ura3-52 His3Δ1 leu2-3,112* | [43] |
| THB0 (A12THB0) | CEN.PK102-5B; pTH-BGL (A12-BGL); pYX242WS | This study |
| BSSO1(A12BSSO1) | CEN.PK102-5B; pTH-BGL (A12-BGL); SSO1 | This study |
| BSNC2(A12BSNC2) | CEN.PK102-5B; pTH-BGL (A12-BGL); SNC2 | This study |
| BSEC1(A12BSEC1) | CEN.PK102-5B; pTH-BGL (A12-BGL); SEC1 | This study |
| BEXO70(A12BEXO70) | CEN.PK102-5B; pTH-BGL (A12-BGL); EXO70 | This study |
| BYPT32(A12BYPT32) | CEN.PK102-5B; pTH-BGL (A12-BGL); YPT32 | This study |
| BSEC4(A12BSEC4) | CEN.PK102-5B; pTH-BGL (A12-BGL); SEC4 | This study |
| BSEC12(A12BSEC12) | CEN.PK102-5B; pTH-BGL (A12-BGL); SEC12 | This study |
| BSEC13(A12BSEC13) | CEN.PK102-5B; pTH-BGL (A12-BGL); SEC13 | This study |
| BERV25(A12BERV25) | CEN.PK102-5B; pTH-BGL (A12-BGL); ERV25 | This study |
| BBOS1(A12BBOS1) | CEN.PK102-5B; pTH-BGL (A12-BGL); BOS1 | This study |
| THC0 (A12THC0) | CEN.PK102-5B; pTH-CEL (A12-CelA); pYX242WS | [47] |
| CSSO1(A12CSSO1) | CEN.PK102-5B; pTH-CEL (A12-CelA); SSO1 | This study |
| CSNC2(A12CSNC2) | CEN.PK102-5B; pTH-CEL (A12-CelA); SNC2 | This study |
| CSEC1(A12CSEC1) | CEN.PK102-5B; pTH-CEL (A12-CelA); SEC1 | This study |
| CEXO70(A12CEXO70) | CEN.PK102-5B; pTH-CEL (A12-CelA); EXO70 | This study |
| CYPT32(A12CYPT32) | CEN.PK102-5B; pTH-CEL (A12-CelA); YPT32 | This study |
| CSEC4(A12CSEC4) | CEN.PK102-5B; pTH-CEL (A12-CelA); SEC4 | This study |
| CSEC12(A12CSEC12) | CEN.PK102-5B; pTH-CEL (A12-CelA); SEC12 | This study |
| CSEC13(A12CSEC13) | CEN.PK102-5B; pTH-CEL (A12-CelA); SEC13 | This study |
| CERV25(A12CERV25) | CEN.PK102-5B; pTH-CEL (A12-CelA); ERV25 | This study |
| CBOS1(A12CBOS1) | CEN.PK102-5B; pTH-CEL (A12-CelA); BOS1 | This study |
| A120 | CEN.PK102-5B; A12; pYX242WS | This study |
| A12SSO1 | CEN.PK102-5B; A12; SSO1 | This study |
| A12SNC2 | CEN.PK102-5B; A12; SNC2 | This study |
| A12CSEC1 | CEN.PK102-5B; A12; SEC1 | This study |
| A12EXO70 | CEN.PK102-5B; A12; EXO70 | This study |
| A12YPT32 | CEN.PK102-5B; A12; YPT32 | This study |
| A12SEC4 | CEN.PK102-5B; A12; SEC4 | This study |
| A12SEC12 | CEN.PK102-5B; A12; SEC12 | This study |
| A12SEC13 | CEN.PK102-5B; A12; SEC13 | This study |
| A12ERV25 | CEN.PK102-5B; A12; ERV25 | This study |
| A12BOS1 | CEN.PK102-5B; A12; BOS1 | This study |

Table S2 The primers used in this study.

| Name | Sequence (5’-3’) |
| --- | --- |
| AGA1-F | CAATCTAATCTAAGTTTTAATTACAAAGGATCCTCTAGAATGACATTATCTTTCGCTC |
| AGA1-R | TTCAATTCAATTCAATCCTGCAGGTCGACTCTAGATTAACTGAAAATTACATTGCAAG |
| BAGA2-F | AATCTAATCTAAGTTTTAATTACAAGCGGCCGCACTAGTATGCAGTTACTTCGCTGTT |
| BAGA2-R | CTTCCAGTGGTAGTGGCTGGGCGGCGGGTGGTGGTGGTGCCACGGTTTCCGCCGGGCCATGGAAAAACATACTGTGTGTTTATGG |
| BGL1-F | ACCACCACCACCCGCCGCCCAGCCACTACCACTGGAAGCTCTCCCGGACCTACCGTCCCAATTCAAAACTATACC |
| BGL1-R | CTTATCGTCGTCATCCTTGTAATCCATCGATACTAGTTCACTTGTCATCGTCGTCCTTG |
| CAGA2-F | TAATTATCTACTTTTTACAACAAATATAAAACAAGGATCCATGCAGTTACTTCGCTGTT |
| CAGA2-R | CTTCCAGTGGTAGTGGCTGGGCGGCGGGTGGTGGTGGTGCCACGGTTTCCGCCGGGGTCGACAAAAACATACTGTGTGTTTATGG |
| CelA-F | CCACCACCACCCGCCGCCCAGCCACTACCACTGGAAGCTCTCCCGGACCTACCGCAGGTGTGCCTTTTAACAC |
| CelA-R | GGATCTTAGCTAGCCGCGGTACCAAGCTTACTCGAGTCACAGATCCTCTTCTGAGATGAG |
| PGK-F | CCCAGTCACGACGTTGTAAAACGACGGCCAGTGAATTCGGAAGTACCTTCAAAGAATGG |
| CYC-R | CGTACAAAGTATGCATTGTGGTACCGAGCTCGAATTCCTTCGAGCGTCCCAAAACCTTC |
| TEF-F | TTGAACGTACAAAGTATGCATTGTGGTACCGAGCTCGCACACACCATAGCTTCAAAATG |
| ADH-R | TCACGACGTTGTAAAACGACGGCCAGTGAATTCGAGCTCGAGCGACCTCATGCTATACC |
| SSO1-F | ATAGCAATCTAATCTAAGTTTTAATTACAAGTCGACATGAGTTATAATAATCCGTACC |
| SSO1-R | CGTTCATTGTTCCTTATTCAGTTAGCTAGCTGAGCTCTTAACGCGTTTTGACAACG |
| SNC2-F | ATAGCAATCTAATCTAAGTTTTAATTACAAGTCGACATGTCGTCATCAGTGCCATAC |
| SNC2-R | AACGTTCATTGTTCCTTATTCAGTTAGCTAGCTGAGCTCTTAGCTGAAATGGACGACG |
| SEC1-F | ATAGCAATCTAATCTAAGTTTTAATTACAAGTCGACATGTCTGATTTAATTGAATTAC |
| SEC1-R | CGTTCATTGTTCCTTATTCAGTTAGCTAGCTGAGCTCTCATTTATCATGGTGAGATT |
| EXO70-F | ATAGCAATCTAATCTAAGTTTTAATTACAAGTCGACATGCCCGCTGAAATTGACATTG |
| EXO70-R | ACGTTCATTGTTCCTTATTCAGTTAGCTAGCTGAGCTCCTATCTCACTAATTGGTTAAG |
| YPT32-F | TAGCAATCTAATCTAAGTTTTAATTACAAGTCGACATGAGCAACGAAGATTACG |
| YPT32-R | ACGTTCATTGTTCCTTATTCAGTTAGCTAGCTGAGCTCTTAACAACAGTTGCTGGAT |
| SEC4-F | TAGCAATCTAATCTAAGTTTTAATTACAAGTCGACATGTCAGGCTTGAGAACTG |
| SEC4-R | ACGTTCATTGTTCCTTATTCAGTTAGCTAGCTGAGCTCTCAACAGCAATTTGATTTAG |
| SEC12-F | ATAGCAATCTAATCTAAGTTTTAATTACAAGTCGACATGAAGTTCGTGACAGCTAG |
| SEC12-R | ACGTTCATTGTTCCTTATTCAGTTAGCTAGCTGAGCTCTCAAGCATCATCTATTTCTCG |
| SEC13-F | ATAGCAATCTAATCTAAGTTTTAATTACAAGTCGACATGGTCGTCATAGCTAATGC |
| SEC13-R | ACGTTCATTGTTCCTTATTCAGTTAGCTAGCTGAGCTCTCACTGATGAACTTCACCAG |
| ERV25-F | ATAGCAATCTAATCTAAGTTTTAATTACAAGTCGACATGCAGGTGTTACAGTTATG |
| ERV25-R | CGTTCATTGTTCCTTATTCAGTTAGCTAGCTGAGCTCTTAAATGATATGTTTCGTTTTG |
| BOS1-F | ATAGCAATCTAATCTAAGTTTTAATTACAAGTCGACATGGTATGTTTGATCGCCG |
| BOS1-R | ACGTTCATTGTTCCTTATTCAGTTAGCTAGCTGAGCTCCTATCTTAACCATTTCAACAC |
